# Esophageal perforation with near fatal mediastinitis secondary to Th3 fracture

**DOI:** 10.1007/s00508-024-02397-3

**Published:** 2024-07-10

**Authors:** Maria Anna Smolle, Alfred Maier, Jörg Lindenmann, Christian Porubsky, Franz-josef Seibert, Andreas Leithner, Freyja-maria Smolle-juettner

**Affiliations:** 1 Department of Orthopaedics and Trauma, Medical University of Graz, Auenbruggerplatz 5, 8036 Graz, Austria; 2 Division of Thoracic and Hyperbaric Surgery, Department of Surgery, Medical University of Graz, Auenbruggerplatz 29/3, 8036 Graz, Austria

**Keywords:** Esophageal injury, Diffuse idiopathic skeletal hyperostosis, Vertebral fracture, Esophagogastrostomy, Trauma

## Abstract

A 74-year-old male patient was referred with signs of sepsis 5 days after having been diagnosed with a rib fracture following a fall out of bed. Novel hypodensities were visible on thoracic X‑rays and laboratory tests revealed elevated inflammatory parameters. Subsequently performed thoracic computed tomography (CT) scan showed burst fracture of the 3rd thoracic vertebra, posttraumatic esophageal rupture at the same level and mediastinitis. Furthermore, marked degenerative changes of the spinal column (diffuse idiopathic skeletal hyperostosis) were present. The patient underwent emergency thoracotomy and esophagectomy. Gastric pull-up with esophagogastrostomy was postponed for 3 days. After 14 days on the intensive care unit (ICU) and 12 days of i.v. antibiotics, the patient was transferred to the general ward and 7 weeks after trauma the patient was infection-free without difficulties in swallowing. Up to the latest follow-up 41 months following injury, several endoscopic dilations with a bougie due to constrictions at the anastomosis have been performed. Similar to previous cases in the literature, esophageal injury was diagnosed delayed, with the patient already having developed severe complications. This extremely seldom injury should be suspected in young patients following high-energy trauma, but also in older patients after low-energy trauma but known degenerative changes of the vertebral column.

## Introduction

In young patients vertebral fractures usually develop due to high-energy trauma, while in older patients with osteoporosis or degenerative changes of the axial skeleton, even minor accidents may lead to fractures of the spine. Fractures most commonly develop in the cervical and lumbar region, whereas those of the upper thoracic level only account for 16% [1]. In this region, the esophagus is in close anatomical contact to the anterior spine, thus being at potential risk of injury [2]. In 1979, the first case of a C4/5 vertebral fracture with associated esophageal injury following a car accident was published [3], with less than 15 reports made public since then [2, 4–9]. The rarity of accompanying esophageal injuries due to vertebral fractures, together with unspecific symptoms and absent radiographic findings, impairs an early diagnosis [4]; however, a delay in treatment of more than 48 h for esophageal rupture increases the mortality rate from 10–25% up to 40–60% [10]. At the same time, these injuries can occur under two nearly opposite circumstances: after high-energy trauma in young patients [3, 5, 6, 11], and following low-energy trauma in older individuals with degenerative changes of the vertebral column (e.g., diffuse idiopathic skeletal hyperostosis, DISH) [8, 12].

## Case presentation

**Fig. 1:**
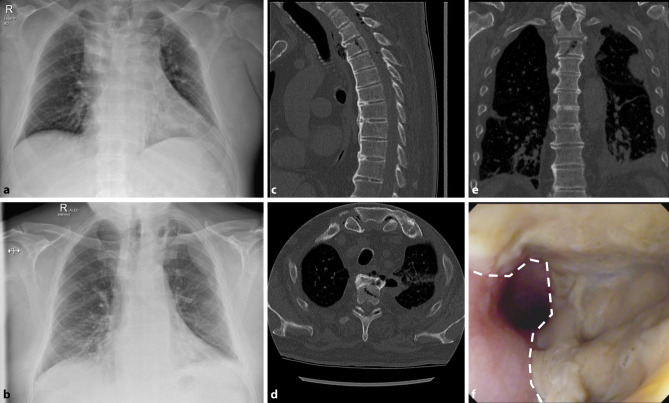
**a** At initial trauma the chest X-ray (frontal plane) taken in a lying position only showed the fracture of the 5th right rib. **b** Areas of encapsulated air and mediastinal widening were visible 5 days later on the chest X‑ray in standing position (frontal plane). Subsequent CT scan in sagittal (**c**), axial (**d**), and frontal (**e**) plane showed an incomplete burst fracture (**c**,**d**,**e**), esophageal perforation (**d**), mediastinitis with fistula (**d**), left-sided pleural empyema (**d**), and entrapped air within the spinal canal (**c**). Endoscopy prior to emergency esophagectomy revealed extensive necrosis involving two thirds of the esophageal wall (**f**; necrosis visible to the right of the dashed line)

A 74-year-old male patient presented to the accident and emergency department after having fallen out of bed and was subsequently diagnosed with a fracture of the 5th right rib and mild concussion based on X‑rays of the thorax (Fig. 1a) and cranial computed tomography (CT, down to level C4). At that time, the patient complained of right-sided thoracic pain on deep inspiration. Known comorbidities included obesity grade I, type II diabetes treated with a sodium-glucose transporter 2 (SGLT-2) inhibitor, hypertension, hepatic steatosis, vitamin D deficiency and sigmoid diverticulosis. Due to an inconspicuous neurological status, the patient was subsequently discharged. Following trauma, the patient’s general practitioner noticed a worsening general condition and progressive difficulties in swallowing. At our outpatient clinic 5 days after trauma, he presented with tachycardia, cold sweat and normothermia (36.7 °C).

Diagnostic X‑rays of the thorax, cervical column and dens axis were taken, with novel hypodensities visible in left upper hemithorax and paramediastinum (Fig. 1b). Laboratory tests revealed leukocytes 7.3 G/L, hemoglobin 13.2 g/dL, creatinine 3.62 mg/dL, estimated glomerular filtration rate (eGFR) 15.58 ml/min, lactate dehydrogenase (LDH) 314 U/L and C-reactive protein (CRP) 484.2 mg/L. As pneumonia was suspected, a thoracic CT scan was performed and in addition to a lateral shaft fracture of the 5th right rib, another fracture of the 5th right rib at the costotransverse joint, a split fracture (AO spine type A2) of the 3rd thoracic vertebra (Fig. 1c, d, e), and signs of DISH, were diagnosed. Most strikingly though, a posttraumatic esophageal rupture at the level of the 3rd thoracic vertebra was diagnosed (Fig. 1d), with concurrent fistula between the mediastinum and pleural cavity (Fig. 1d), loculated left-sided pleural empyema, and entrapped air within the spinal canal (Fig. 1c). Notably, due to the patient’s serious general condition, a magnetic resonance imaging (MRI) scan to rule out injury of intervertebral discs, posterior structures, or the presence of accompanying spondylitis, was not carried out.

Blood cultures were obtained prior to the start of empirical i.v. antibiotic therapy with teicoplanin (1 × 1.2 g), clindamycin (3 × 600 mg) and piperacillin/tazobactam (3 × 4.5 g). Subsequent endoscopy showed widespread necrosis of the esophageal mucosa (Fig. 1f). The patient underwent emergency thoracotomy, revealing necrotizing mediastinitis, mediastinal abscess and loculated empyema due to a large defect in the esophagus and necrosis of two thirds of its wall caused by perforation due to a small vertebral fragment. Thus, the esophagus could not be preserved and emergency esophagectomy as well as mediastinal and pleural debridement were performed. Reconstruction by retrosternal gastric pull-up with cervical esophagogastrostomy had to be postponed for 3 days owing to the patient’s critical condition with sepsis, renal failure requiring hemodialysis and cardiorespiratory instability.

**Fig. 2:**
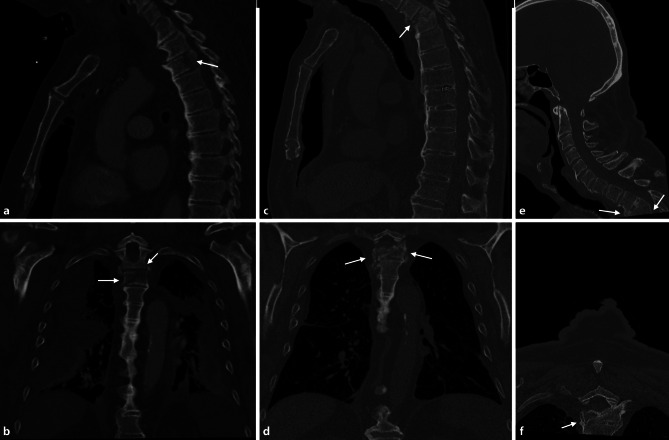
Follow-up images showing no apparent fracture healing at 18 days following trauma (thoracic CT scan; **a** sagittal plane, ** b** frontal plane), progressive destruction of the T3 vertebra at 3 months (thoracic CT scan; **c** sagittal plane, **d** frontal plane), and finally intersegmental fusion after 41 months (cranial CT scan down to cervicothoracic junction; **e** sagittal plane, **f** axial plane). *Arrows* mark T3 vertebra

The blood cultures came back positive for *Streptococcus anginosus* 3 days after admission (sensitive to penicillin, ampicillin, cefuroxime, piperacillin/tazobactam, cefazolin, amoxicillin/clavulanic acid, cefotaxime, ceftriaxone, cefepime, imipenem, meropenem, ertapenem, vancomycin, teicoplanin; resistant to clindamycin) and antibiotic therapy was adapted to piperacillin/tazobactam monotherapy (3 × 4.5 g). Weaning from the ventilator was difficult and required temporary tracheostomy. Due to the non-dislocated nature of the vertebral fracture, lack of neurological impairment, the patient’s initially critical general condition and potentially contaminated future surgical area, no surgical stabilization was performed. Antibiotic therapy was discontinued after 12 days due to declining inflammatory parameters. After 14 days on the ICU, the patient was transferred to the general ward and was discharged in a fair general condition 7 weeks after the trauma, with unimpaired neurological status, no restrictions in swallowing liquids or food, and without signs of infection. Follow-up CT images at 18 days, 3 months and 41 months revealed a progressive destruction of T3, and finally intersegmental fusion (Fig. 2). Up to the latest follow-up 41 months after injury, the patient has required several endoscopic dilations with a bougie due to constrictions at the anastomosis with consecutive dysphagia, but is otherwise free of complaints, with no back pain or neurological deficits recorded that would be indicative of instability or spinal stenosis.

## Discussion

Fractures at levels T3 and T4 appear to be at higher risk to cause concomitant esophageal injuries than vertebral fractures at other segments, considering that most cases in the literature so far reported on vertebral fractures at this level [2, 4–9]. So far, 11 case reports have been published on vertebral fracture following trauma with associated esophageal injury (Table 1; [2–9, 11–13]). All but one [13] reported on distraction and/or translation injuries, whereas in our study, a split fracture was diagnosed on CT scan. Notably, as no MRI images were carried out owing to the patient’s critical general condition, the presence of a more unstable fracture type cannot be ruled out with certainty. Although high-energy driving accidents seem to be the prevailing cause of esophageal injury secondary to vertebral fractures [2–7, 9, 11, 13], our patient had experienced relatively mild trauma. Of note, in these cases, degenerative changes (e.g., due to ankylosing spondylitis) of the vertebral column are usually present [8, 12], similar to our patient’s DISH. Additional features potentially associated with esophageal injury secondary to vertebral fracture have not been described so far.

**Table 1:** Case list of esophageal injury secondary to vertebral fractures published in literature

Author	Age (years), gender	Trauma	Comorbidities	Diagnostic delay of esophageal injury	Symptoms	Spinal injury	Esophageal injury	Treatment
Present study	*74, M*	*Fall*	*DISH, diabetes type II, obesity, hepatic steatosis, vitamin D-deficiency, sigmoid diverticulosis*	*6 days*	*Day 0: thoracic pain*	*T3 split fracture (A2)*	*Esophageal perforation*	*Day 6: endoscopy, emergency thoracotomy, esophagectomy, mediastinal and pleural debridement; i.v. antibiotics*
					*Day 6: septicemia, difficulties swallowing*			*Day 9: Retrosternal gastric pull-up*
Makoyo PZ 1979 J Nat Med Assoc [3]	22, M	Driving accident	None reported	4 days	Day 0: Quadriplegia (from C5)	C4/5 fracture-dislocation	Esophageal perforation	Day 0: Halo traction
					Day 4: Fever			Day 4: Drainage of retroesophageal space; gastrostomy; i.v.-antibiotics; posterior mediastinotomy and drainage
Maroney MJ 1996 AJR [4]	58, M	Driving accident	None reported	None	Day 0: Back pain, bilateral lower extremity numbness, dysphagia	T3/4 fracture-dislocation	Esophageal entrapment (between T3 and T4)	Day 0: Thoracotomy, ORIF, esophageal occlusion, feeding gastrostomy;
								Week 5: Secondary esophageal re-anastomosis
Brouwers MA 1997 Eur Spine J [5]	17, M	Motorcycle accident	None reported	14 days	Day 0: Brown-Sequard syndrome (from T6)	T4 (type C3.1)	Esophageal perforation	Day 0: Halo traction
								Day 14: Anterior stabilization of fracture with Slot-Zielke device
								for 10 weeks: Feeding via jejunostomy
Nakai S 1998 J Trauma [2]	48, F	Motorbike accident	None reported	11 days	Day 0: Back pain, respiratory difficulties	T3/4 fracture dislocation	Eosophageal perforation	Conservative (chest drains, povidone iodine lavage of mediastinum)
					Day 3: Fever, worsening respiratory situation			
Chen SH 2002 J Bone Joint Surg Am [6]	20, M	Motorbike accident	None reported	None	Day 0: Thoracostomy yielding abundant air, blood and food debris	Oblique shear fracture T4	Tracheoesophageal perforation	Day 0: Closure of tracheal and esophageal tear, coverage with intercostal muscular flap via thoracotomy
								Day 5: Posterior spinal stabilization
Chen HC 2005 Acta Neurochir (Wien) [11]	49, M	Motorbike accident	None reported	7 days	Day 0: Hyperesthesia and motor weakness (grade 3/5); Day 7: Upper back and abdominal pain	Disruption C5/6 anterior longitudinal ligament; C3/4 and C5/6 disc herniation; T1 compression fracture	Esophageal perforation	Day 0: Anterior discectomy (C3/4, C4/5, C5/6), bone grafts and Caspar plate fixation Day 7: Feeding jejunostomy tube Day 13: emergency laminectomy and abscess drainage
Tjardes T 2009 Eur Spine J [7]	58, M	High-velocity driving accident	None reported	6 days	Day 0: No neurological deficits	T3/4 hyperextension-type fracture	Esophageal rupture at T3/4	Day 0: Percutaneous spinal stabilization
					Day 6: Fever, septicemia			Day 11: Esophageal stenting
Lee DH 2011 Spine J [13]	49, M	Driving accident	None reported	None	Day 0: Paraplegia (grade 2/5)	Day 0: T2 compression fracture	Esophageal entrapment	Day 2: Laminoplasty C3–C6
					5 months: Intermittent fever	5 months: Spondylodiscitis T1–T3		5 months: i.v.-antibiotics, neck brace
Delappe RS 2013 Emerg Radiol [8]	67, F	Fall	End stage renal disease, type II diabetes	4 days	Day 0: None	C5 burst fracture, T3/4 transverse fracture	Esophageal entrapment at level T3/4	Initially: C5 corporectomy, C4–C6 anterior fusion; feeding gastrostomy
					Day 4: Neck pain, headache			
Groen FRJ 2016 Eur Spine J [9]	73, M	Driving accident	Ankylosing spondylitis	None	Day 0: Severe back pain, difficulties swallowing	T4–T6 hyperextension type fracture	Esophageal rupture at T3/4	Initially: Percutaneous spinal stabilization, primary suture of esophagus
Vonhoff, CR 2018 World Neurosurg [12]	66, M	Fall	Ankylosing spondylitis	7 days	Day 0: Back pain	C6 transverse fracture	Esophageal entrapment (within C6)	Day 7: Anterior plate and posterolateral mass/screw fixation; primary esophageal suture
					Day 5: Dysphasia, difficulties clearing oral secretion			

*ORIF *open reduction internal fixation

As previously reported [2, 3, 7, 8, 11–13] symptoms indicative of esophageal injury, such as dysphagia or vomiting were lacking in our patient at initial presentation. Supposedly, the accompanying rib fracture may have masked pain actually related to the vertebral fracture, wherefore further diagnostic work-up despite thoracic X‑ray showing a reasonable injury was omitted. Unsurprisingly, pneumonia was suspected when the patient presented 5 days after trauma with septicemia. Thoracic CT scan did not only reveal signs of mediastinitis with peri-esophageal air entrapment, but also trapped air within the spinal canal, a feature indicative of esophageal rupture [4]. Due to the rarity of esophageal perforation secondary to vertebral fracture, and either masking by other injuries following high-energy trauma (e.g., long bone fractures, pneumothorax, brain injury) or low-energy trauma rendering severe internal organ injuries unlikely, the diagnosis was likewise delayed in most previous cases [2, 3, 5, 7, 11–13].

In line with other reports [3–5, 9] multidisciplinary management including fast and extensive surgical debridement, esophagectomy, i.v. antibiotic therapy, and secondary reconstruction via gastric pull-up was essential to save the patient’s life. Herein, antibiotic therapy was discontinued after 12 days due to decreasing inflammatory parameters. In retrospect, this can be seen critically given the anatomical proximity of the esophageal perforation and vertebral fracture, and thus a high risk for subsequent spondylitis. Nevertheless, apart from repetitive endoscopic dilations due to esophageal constrictions, the patient did not develop any further complications up to the latest clinical visit.

## Conclusion

Despite the rarity of esophageal injury secondary to upper thoracic vertebral fracture, it should be considered in young patients following high-energy trauma and in older patients with marked degenerative changes of the spine. This constellation should prompt further diagnostic work-up, including thoracic CT scans. In cases where a diagnostic delay has led to mediastinitis, aggressive surgery rather than attempts at conservative treatment is inevitable to save the patients’ life.
